# CXCL13 expressed on inflamed cerebral blood vessels recruit IL-21 producing T_FH_ cells to damage neurons following stroke

**DOI:** 10.1186/s12974-022-02490-2

**Published:** 2022-05-27

**Authors:** Aditya Rayasam, Julie A. Kijak, Lee Kissel, Yun Hwa Choi, Taehee Kim, Martin Hsu, Dinesh Joshi, Collin J. Laaker, Peter Cismaru, Anders Lindstedt, Krisztian Kovacs, Raghu Vemuganti, Shing Yan Chiu, Thanthrige Thiunuwan Priyathilaka, Matyas Sandor, Zsuzsanna Fabry

**Affiliations:** 1 Department of Pathology and Laboratory Medicine, School of Medicine and Public Health, University of Wisconsin-Madison, Madison, WI USA; 2 Neuroscience Training Program, University of Wisconsin-Madison, Madison, WI USA; 3 School of Pharmacy, University of Wisconsin-Madison, Madison, WI USA; 4 Department of Neurological Surgery, University of Wisconsin, Madison, WI USA; 5 Department of Physiology, University of Wisconsin School of Medicine, Madison, WI USA; 6 Cellular and Molecular Pathology Graduate Program, University of Wisconsin-Madison, Madison, WI USA; 7 William S. Middleton Memorial Veterans Administration Hospital, Madison, WI USA

**Keywords:** Lymphocytes, Neuroinflammation, Stroke, Immune, Brain, IL-21, T cells, Neurons, Apoptosis, MCAO

## Abstract

**Background:**

Ischemic stroke is a leading cause of mortality worldwide, largely due to the inflammatory response to brain ischemia during post-stroke reperfusion. Despite ongoing intensive research, there have not been any clinically approved drugs targeting the inflammatory component to stroke. Preclinical studies have identified T cells as pro-inflammatory mediators of ischemic brain damage, yet mechanisms that regulate the infiltration and phenotype of these cells are lacking. Further understanding of how T cells migrate to the ischemic brain and facilitate neuronal death during brain ischemia can reveal novel targets for post-stroke intervention.

**Methods:**

To identify the population of T cells that produce IL-21 and contribute to stroke, we performed transient middle cerebral artery occlusion (tMCAO) in mice and performed flow cytometry on brain tissue. We also utilized immunohistochemistry in both mouse and human brain sections to identify cell types and inflammatory mediators related to stroke-induced IL-21 signaling. To mechanistically demonstrate our findings, we employed pharmacological inhibitor anti-CXCL13 and performed histological analyses to evaluate its effects on brain infarct damage. Finally, to evaluate cellular mechanisms of stroke, we exposed mouse primary neurons to oxygen glucose deprivation (OGD) conditions with or without IL-21 and measured cell viability, caspase activity and JAK/STAT signaling.

**Results:**

Flow cytometry on brains from mice following tMCAO identified a novel population of cells IL-21 producing CXCR5+ CD4+ ICOS-1+ T follicular helper cells (T_FH_) in the ischemic brain early after injury. We observed augmented expression of CXCL13 on inflamed brain vascular cells and demonstrated that inhibition of CXCL13 protects mice from tMCAO by restricting the migration and influence of IL-21 producing T_FH_ cells in the ischemic brain. We also illustrate that neurons express IL-21R in the peri-infarct regions of both mice and human stroke tissue in vivo*.* Lastly, we found that IL-21 acts on mouse primary ischemic neurons to activate the JAK/STAT pathway and induce caspase 3/7-mediated apoptosis in vitro*.*

**Conclusion:**

These findings identify a novel mechanism for how pro-inflammatory T cells are recruited to the ischemic brain to propagate stroke damage and provide a potential new therapeutic target for stroke.

## Introduction

Stroke is one of the leading causes of death and disability worldwide. Despite ongoing intensive research, there has been limited translational success of pharmacological interventions and the only FDA approved therapy remains to be tissue plasminogen activator (tPA) [1]. One of the challenges of developing stroke therapies is the myriad of physiological mechanisms that occur within the patient following the disease, which include excitotoxicity, edema and inflammation [2–5]. Another challenge is the heterogeneous pathology from patient to patient due to the influence of age, sex, and other epidemiological heterogeneities [6–9].

While stroke incidence could be due to the hemorrhage of cerebral blood vessels, 87% of strokes are due to artery occlusions, which induce brain ischemia [6]. During ischemic stroke, occlusion of cerebral blood vessels deprives the brain of its required oxygen and nutrients to elicit the primary brain damage. Furthermore, once the occlusion is bypassed, peripheral blood containing pro-inflammatory immune cells reperfuse the once-occluded vessels to create secondary damage, which can oftentimes be more harmful than the occlusion-mediated damage itself. Several laboratories have attempted to identify the major pro-inflammatory cells and molecules responsible for most of the secondary tissue injury that occurs during the reperfusion phase of ischemic stroke [10–13].

Due to the application of different animal models and immune cell depletion strategies, controversies have emerged regarding the influence of how immune cell subtypes mediate ischemic brain injuries [14–20]. T cells have been identified to have a major role in ischemic stroke pathology. This is supported by data indicating that mice deficient in lymphocytes have altered brain pathology and cognitive functions following ischemic injury [13, 20, 21]. Depending on the timing of their involvement and phenotype, T cells can either be beneficial or detrimental to stroke outcome. For example, regulatory T cells have been shown to be beneficial in stroke regeneration, partly due their production of IL-10 [13–17]. Moreover, the neuroprotective effects of IL-33 have been associated with its role in promoting the differentiation of anti-inflammatory TH_2_ cells [18].

Alternatively, we and others have identified that certain phenotypes of T cells can be recruited to the ischemic brain during the acute phase of stroke to augment brain damage. For example, IL-17-producing nonconventional γδ T cells have been implicated in the recruitment of neutrophils and promoting ischemic brain damage [11, 19, 20]. Furthermore, perforin, which is a pore forming cytolytic protein produced by cytotoxic T lymphocytes (CTLs) and NK cells, has also been implicated in potentiating stroke as well [21]. During the acute phase of ischemic stroke, our lab previously demonstrated that CD4 T cell-produced IL-21 contributes to damage following transient middle cerebral artery occlusion (tMCAO) in mice and postmortem brain tissues from stroke patients contain IL-21+ cells in perivascular regions in the necrotic brain parenchyma [22]. This raised the possibility that understanding how IL-21 producing T cells enter the ischemic brain could reveal novel therapeutic approaches for stroke.

T follicular helper (T_FH_) cells are a unique subset of helper T cells that produce IL-21. While most of these cells are present in the peripheral lymph nodes, exciting recent data shed light on the association with infiltrating T_FH_ and exacerbated clinical outcome of numerous diseases spanning from autoimmunity to immunodeficiency. The expression of CXCR5 on T_FH_ cells is associated with autoimmune disease progression [23] and serum levels of CXCR5 on T_FH_ cells are associated with aggravated rheumatoid arthritis [24]. Data suggest that this chemokine receptor facilitates the ability of circulating T_FH_ cells to rapidly extravagate passed the BBB into inflamed tissues [25] and serve as a core sentinel of dysregulated immunity in their target tissue [26]. Evidence of these T_FH_ cells migrating into the inflamed brain after ischemic injury might play an important role in acute tissue damage, the formation of ectopic lymphoid structures, and subsequent long-term inflammation following stroke.

The ligand for CXCR5, CXCL13, has been demonstrated to play a role in mediating neuroinflammation and its expression has been observed in the ischemic brain following tMCAO in mice [27–30]. Nonetheless, the mechanism for how the CXCL13-CXCR5 axis contributes to ischemic stroke is unknown. Here, we show that CXCL13 is upregulated on inflamed vascular cells, and that during reperfusion, IL-21 producing CD4+ ICOS-1+ CXCR5+ T_FH_ cells are recruited to the brain where they can induce caspase-mediated neuronal death and potentiate secondary stroke damage.

## Materials and methods

### Mice

C57BL/6 WT mice were obtained from Jackson Laboratory. All mice underwent SHAM or 1-h occlusion and 4-h or 24-h reperfusion. All animal procedures used in this study were conducted in strict compliance with the Guide for the Care and Use of Laboratory Animals (U.S. Department of Health and Human Services) and in compliance with the ARRIVE guidelines (Animal Research: Reporting in Vivo Experiments). All research was approved by the University of Wisconsin Center for Health Sciences Research Animal Care Committee. All mice (∼ 25 g) were anesthetized with 5% halothane for induction and 1.0% halothane for maintenance vaporized in N_2_O and O_2_ (3:2) and all efforts were made to minimize suffering. Male and female mice between 10 and 14 weeks were used for all studies.

### Regional cerebral blood flow (rCBF) measurement

Changes in rCBF at the surface of the left cortex were recorded using a blood perfusion monitor (Laserflo BPM2; Vasamedics) with a fiber optic probe (0.7 mm diameter). The tip of the probe was fixed with glue on the skull over the core area supplied by the MCA (2 mm posterior and 6 mm lateral from bregma). Changes in rCBF after MCAO were recorded as a percentage of the baseline value. Mice included in these investigations had > 80% relative decrease in rCBF during MCAO.

### Investigation of intracranial vasculature

Mice were anesthetized with ketamine (100 mg/kg, i.p.) and xylazine (10 mg/kg, i.p.). After thoracotomy was performed, a cannula was introduced into the ascending aorta through the left ventricle. Transcardial perfusion fixation was performed with 2 ml saline and 2 ml of 3.7% formaldehyde. Carbon lampblack (C198-500; Thermo Fisher Scientific) in an equal volume of 20% gelatin in ddH_2_O (1 ml) was injected through the cannula. The brains were removed and fixed in 4% PFA overnight at 4 °C. Posterior communicating arteries (PComA) connect vertebrobasilar arterial system to the Circle of Willis and internal carotid arteries, and its development affects brain sensitivity to ischemia among different mouse strains [31]. Development of PComA in both hemispheres was examined and graded on a scale of 0–3, as reported previously [32]: 0, no connection between anterior and posterior circulation; 1, anastomosis in capillary phase (present but poorly developed); 2, small truncal PComA; 3, truncal PComA.

### Focal ischemia model

Focal cerebral ischemia in mice was induced by occlusion of the left MCA, as described previously [33]. Operators performing surgeries were masked to experimental groups. In brief, the left common carotid artery was exposed, and the occipital artery branches of the external carotid artery (ECA) were isolated and coagulated. After coagulation of the superior thyroid artery, the ECA was dissected distally and coagulated along with the terminal lingual and maxillary artery branches. The internal carotid artery (ICA) was isolated, and the extracranial branch of the ICA was then dissected and ligated. A standardized polyamide resin glue-coated 6.0 nylon monofilament (3021910; Doccol Corp) was introduced into the ECA lumen, and then advanced ∼ 9 to 9.5 mm in the ICA lumen to block MCA blood flow. During the entire procedure, mouse body temperature was kept between 37 and 38 °C with a heating pad. The suture was withdrawn 60 min after occlusion. The incision was closed, and the mice underwent recovery.

### Infarct measurement

Following tMCAO, animals were killed and trans-cardiac perfusion with 4% phosphate-buffered paraformaldehyde (PFA) was performed. Each brain was postfixed, cryoprotected, and sectioned (coronal; 30 μm thickness at an interval of 320 μm). Serial sections were stained with cresyl violet, scanned and analyzed using the ImageJ software (National Institutes of Health). Ischemic infarct volume was calculated by numeric integration of data from five serial coronal sections in respect to the sectional interval as described previously [34, 35]. Each infarct volume was corrected to account for edema and differential shrinkage during tissue processing using the Swanson formula [36].

### Lymphocyte isolation, and cytofluorometry (FACS)

Mice were deeply anesthetized with isoflurane and then transcardially perfused with cold PBS. Single cell suspensions were made from cervical lymph nodes (CLNs) and spleens by grinding the tissues between the frosted ends of glass slides [37]. Red blood cells were lysed using ACK lysis buffer, and cells were washed with HBSS. Brain and spinal cord tissues were minced with razor blades and pushed through 70-μm nylon cell strainers. Cells were washed, resuspended in 70% Percoll and overlaid with 30% Percoll. The gradient was centrifuged at 2400 rpm for 30 min at 4 °C without brake. The interface was removed and washed before plating. All collected organs were weighed, and live cells were counted using a hemocytometer. Data were acquired on a BD LSR II flow cytometer (BD Biosciences) and analyzed using FlowJo software (Tree Star, Inc., Ashland, OR).

### Fluorescent microscopy

For frozen sections, mice were first perfused with cold PBS, followed by perfusion with 4% PFA/PBS. Harvested tissues were left in 25% sucrose/PBS overnight at 4 °C. 10- to 40-μm-thick tissue cryosections were cut and stored at − 80 °C until staining. Floating sections were incubated in PBS 2 times for 10 min at room temperature before applying primary conjugated antibodies in FACS buffer (PBS/ with 2% BSA/ and 0.1% sodium azide) with 0.1% Triton X-100 (1:1000) overnight at 37 °C. Sections were then washed 2 times for 10 min each time with PBS and secondary antibodies were applied in PBS (1:500) for 2 h if necessary. Lastly, sections were washed 3 times for 10 min each time with PBS and mounted with ProLong Gold antifade reagent containing DAPI (Invitrogen). All images were acquired with a camera (Optronics Inc., Goleta, CA) mounted on a fluorescence microscope (Olympus BX41, Leeds Precision Instruments). The brightness/contrast of the acquired digital images was applied equally across the entire image and equally to control images and analyzed using Adobe Photoshop CS4 software (Adobe Systems Inc., San Jose, CA).

### Antibodies

The following fluorophore-conjugated antibodies were purchased from BD Biosciences (Franklin Lakes, NJ): anti-CD4 (RM4-5), anti-ICOS-1 (D10.G4.1), anti-CXCR5 (2G8). All isotype controls were purchased from BD Biosciences. Anti-Fcγ-R (2.4G2) was produced from a hybridoma. IBA-1(019-19741) was purchased from WAKO (Richmond, VA). Anti-MAP2 (MAB8304) and anti-CXCL13 (AF470) were purchased from R and D systems (Minneapolis, MN). Anti-GFAP (AB5541), Anti-NeuN (MAB377B) and Anti-IL-21 (06-1074) was purchased from EDM Millipore (Hayward, CA). Anti-IL-21R (PA5-19982) was purchased from Invitrogen (Carlsbad, CA). JAK/STAT Phospho Antibody Array was purchased from Full Moon BioSystems (Sunnyvale, CA).

### Oxygen glucose deprivation

Primary neuronal cultures derived from embryonic day 14–18 mouse cortices were grown to 80% confluency in neural basal media supplemented with B27 (2%) and penicillin/streptomycin (1%), as previously described [38]. Astrocytic and microglial contamination was excluded based on the absence of GFAP^+^ and CD11b^+^ cells when stained by immunocytochemistry. For OGD, media were replaced with neural basal media with or without glucose and placed in a hypoxic chamber or under normoxic conditions for 90 min at 37 °C.

### Human tissue samples

Unidentified human brain samples were received from the Department of Pathology tissue bank and were exempt by IRB. Pathologist Dr. Krisztian Kovacs evaluated these acute human stroke tissue samples. Four separate acute infarct cases, using 10 tissues sections/case were analyzed and one representative case is shown. Patients all had acute infarcts: Case 1: 59-year-old female with 24 h acute infarct; 2: 50-year-old woman, 3 days acute infarct; 3: 57-year-old man, less than 3 days acute infarct; 4: 55-year-old woman, 5 days acute infarct.

### Statistical analyses and quality standards

All surgeries were performed in a blinded manner and measurements masked where possible. Infarct volume measurements from cresyl violet-stained sections were averaged from multiple independent blinded observers. Based on power calculations, *n* = 3–10 sex- and age-matched mice were used for each experiment and group assignment was randomized. Among animals receiving tMCAO procedure, 83% of WT mice were included in analysis as 17% displayed no injury. Mice were excluded due to premature death or vessel variation. Results are given as means ± s.e.m. Multiple comparisons were made using one-way ANOVA. Where appropriate, two-tailed nonparametric Mann–Whitney *U* test analysis was used for comparing measures made between two groups. *P*-values < 0.05 were considered significant. *P < 0.05, **P < 0.01, ***P < 0.001, ****P < 0.0001. *n.s.* not significant.

## Results

### T follicular helper cells infiltrate the ischemic brain

The combination of CD4, ICOS-1, CXCR5, and IL-21 is a signature of T follicular helper cells (T_FH_), which have been identified to play an important role in helping B cells produce antibodies within B cell follicles of secondary lymph organs such as the lymph nodes, spleen and Peyer’s patches [39, 40]. Nonetheless, while T_FH_ cells have been implicated to be involved in the pathology of CNS autoimmune diseases [40–43] and spinal cord pathologies [44, 45]; to our knowledge, T_FH_ cells have never been identified in the ischemic brain. Previously, we have found that IL-21 producing T_FH_ cells affect post-stroke recovery [22] but had not comprehensively defined their phenotype. To determine whether these IL-21 producing T cells were T_FH_ cells_,_ we sought to identify these cells comprehensively via flow cytometry [42, 46].

**Fig. 1:**
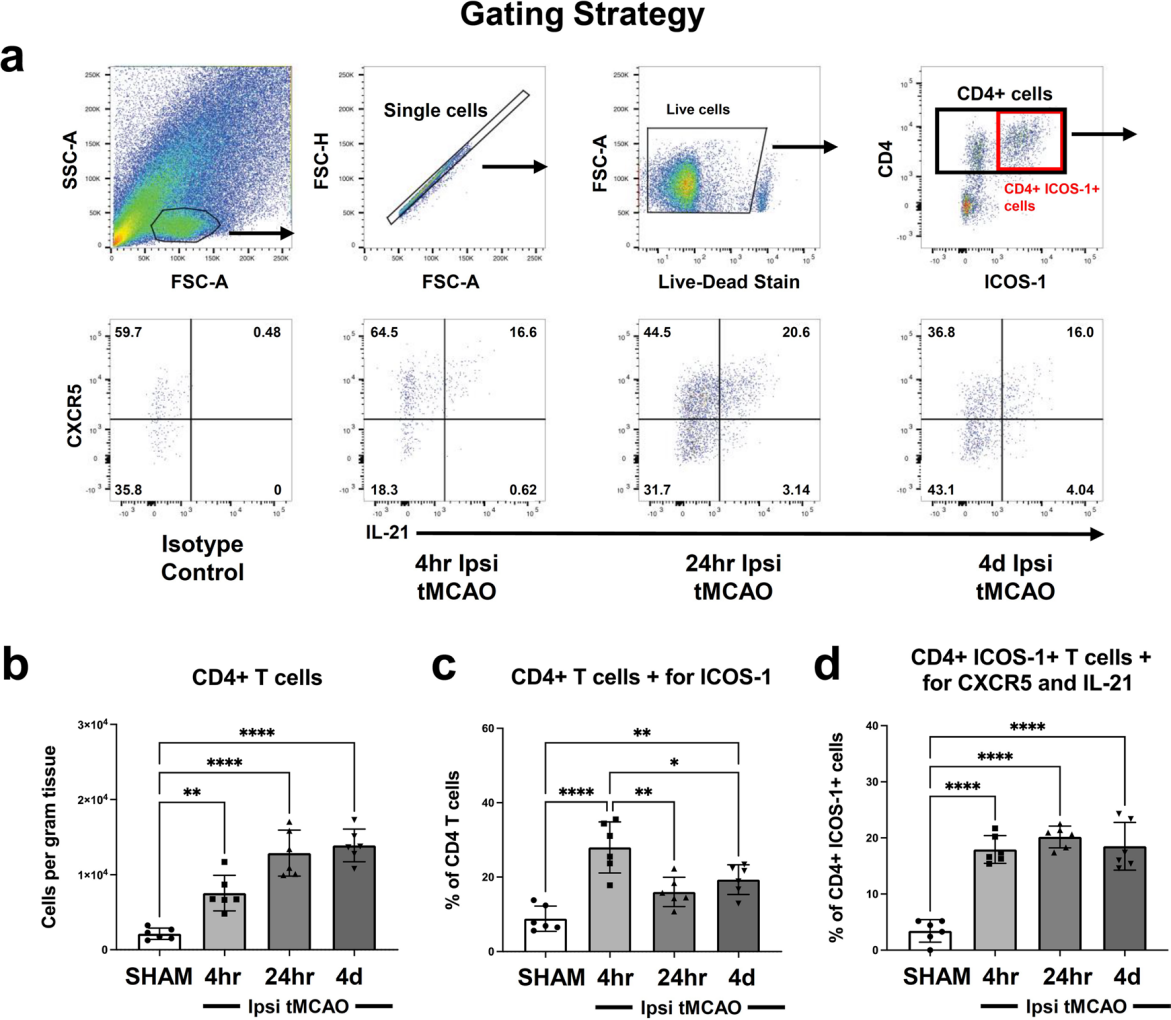
T_FH_ cells infiltrate the ischemic brain. **a** Representative gating from ipsilateral brains harvested from SHAM mice, 4 h, 24 h, and 4 days post-tMCAO in WT mice. Following gating on single cells and live cells, we subsequently gated on cells double-positive for CD4 and ICOS-1. From the double-positive CD4+ ICOS-1+ cells, CXCR5 and IL-21 was gated based on isotype control for IL-21. **b**–**d** Quantification of CD4+ T cells **(b)**, percentage of CD4 T cells positive for ICOS-1 (**c**), and % of CD4+ ICOS-1+ T cells positive for both CXCR5 and IL-21 (**d)** in ipsilateral brains of SHAM mice and mice 4 h, 24 h, and 4 days post-tMCAO (*n* = 6). Data represent mean ± s.e.m. *P < 0.05, **P < 0.01, ***P < 0.001, ****P < 0.0001. *n.s.* not significant. Data are combined from three independent experiments. One-way ANOVA followed by Dunn’s post hoc test (**b**–**d**)

We first performed flow cytometry on isolated ipsilateral brain cells from SHAM operated mice and mice 4 h, 24 h and 4 days post-tMCAO and identified a population of CD4+ ICOS-1+ cells. On those double-positive cells, we then gated on CXCR5 and for intracellular expression of IL-21 (Fig. 1a). Identification and quantification of the number of cells per gram tissue in brains harvested from SHAM and tMCAO mice revealed that CD4+ CXCR5+ ICOS-1+ IL-21+ cells are rapidly recruited to the ischemic brain within 4 h after injury and are maintained in the ischemic brain up to 4 days (Fig. 1b). The percentage of CD4+ ICOS-1+ cells that also expressed CXCR5+ and IL-21+ were also maintained up to 4 days after injury yet seemed to peak early after tMCAO (Fig. 1c, d). These data highlight that CD4+ CXCR5+ ICOS-1+ IL-21+ cells are present in the ischemic brain post-tMCAO and reveal their dynamics for entry into the ischemic brain.

### Cerebral blood vessels upregulate ICAM-1 and express CXCL13 in the ipsilateral brain hemisphere after tMCAO

**Fig. 2:**
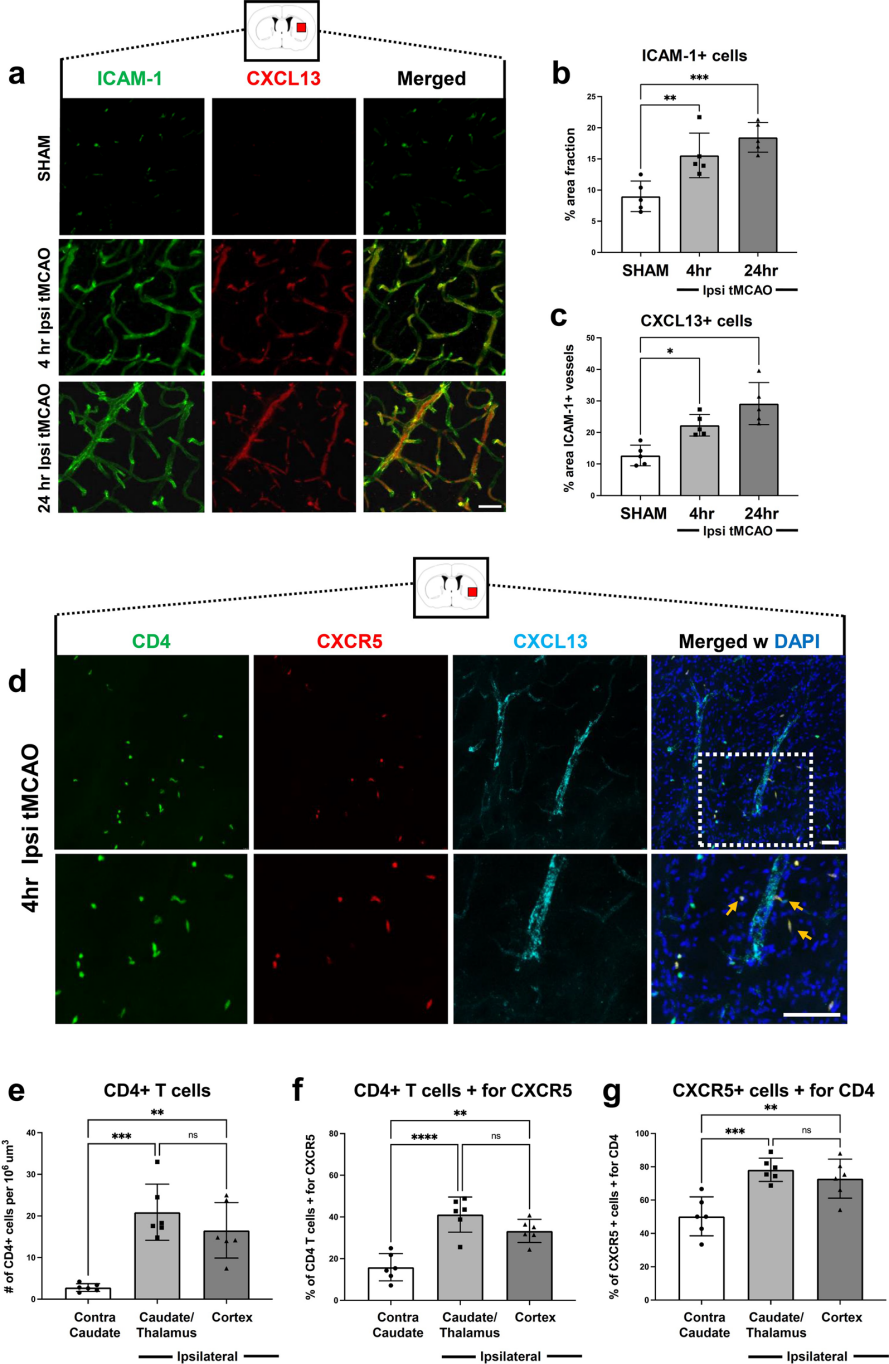
Increased expression of CXCL13 on ICAM-1^+^ cerebral blood vessels leads to accelerated CD4 + CXCR5 + cell infiltration in the ipsilateral brain hemisphere after tMCAO. **a** Representative images of immunohistochemistry (IHC) staining of mouse brains with anti-ICAM-1 (green), and anti-CXCL13 (red) antibodies, and merged image (right panel) of cerebral blood vessels in ipsilateral brain hemisphere following SHAM conditions, as well as at 4 h and 24 h post-tMCAO. **b** Quantification of ICAM-1+ area in ipsilateral brain hemisphere following SHAM conditions, as well as at 4 h and 24 h post-tMCAO (*n* = 5). **c** Quantification of percentage area of ICAM-1+ vessels that are also CXCL13+ in ipsilateral brain hemisphere following SHAM conditions, at 4 h post-tMCAO and at 24 h post-MCAO in mice (*n* = 5). **d** Representative low (top panels) and high (bottom panels) magnification images of CD4 (green), CXCR5 (red), and CXCL13 (cyan) IHC staining at the peri-infarct area, 4 h post-tMCAO. Bottom panels are a higher magnification image of the dotted box from the top panel to highlight double-positive CD4+ CXCR5+ T cells indicated by orange arrows. Scale bar = 30 μm. **e–g** Quantification of CD4+ T cells (**e**), percentage of CD4+ T cells positive for CXCR5 (**f**), and percentage of CXCR5+ cells positive for CD4 (**g**) in the contralateral caudate, ipsilateral caudate/thalamus and ipsilateral cortex. Data are combined from three independent experiments. Data represent mean ± s.e.m. *P < 0.05, **P < 0.01, ***P < 0.001, ****P < 0.0001. n.s. = not significant. One-way ANOVA followed by Dunn’s post hoc test (**b, c, e, f, g**)

To understand the mechanism for how T_FH_ cells are recruited to the ischemic brain, we stained for CXCL13, the cognate ligand for CXCR5. Previously reports have identified that CXCL13 is expressed on ischemic blood vessels within the brain after stroke [30]. Here, we identified that cerebral vessels upregulate Intracellular Adhesion Molecule 1 (ICAM-1) and express CXCL13 4 h and 24 h post-tMCAO (Fig. 2a). Quantifications indicate that ICAM-1 expression is upregulated at 4 and 24 h post-tMCAO compared to SHAM in the peri-infarct area (Fig. 2b). Furthermore, co-localization of ICAM-1 and CXCL13 is also increased at both 4 h and even more so at 24 h post-tMCAO compared to SHAM (Fig. 2c). These data reveal that cerebral blood vessels up-regulate ICAM-1 and CXCL13 in the ipsilateral hemisphere 24 h post-tMCAO, revealing a potential mechanism for how CXCR5+ IL-21 producing T_FH_ cells are recruited to the ischemic brain.

We also assessed whether CXCR5+ CD4+ T cells were in the vicinity of CXCL13 expressing vessels following tMCAO. We observed abundant CD4 T cells in the peri-infarct region 4 h after tMCAO with many of them also expressing CXCR5. The bottom row is a magnified image of the image in the top row to highlight double-positive CD4+ CXCR5+ T cells indicated by orange arrows (Fig. 2d). Moreover, we quantified the number of CD4 T cells (Fig. 2e) and the percentage of CD4 T cells positive for CXCR5 (Fig. 2f) in the contralateral caudate as well as the ipsilateral caudate/thalamus and cortex to demonstrate that a large proportion of CD4+ T cells (~ 30 to 40%) express CXCR5 in the ischemic hemisphere at this time point. Lastly, given that other immune cells can express CXCR5 [47], we quantified the percentage of CXCR5+ cells that were positive for CD4 (Fig. 2g) to illustrate that a majority of the cells expressing that receptor are CD4+ T cells. Altogether, these data demonstrate that CD4+ CXCR5+ T cells are present in the ischemic brain early after injury and are associated with CXCL13+ vessels.

### Administration of anti-CXCL13 antibody reduces infarct volume following tMCAO

**Fig. 3:**
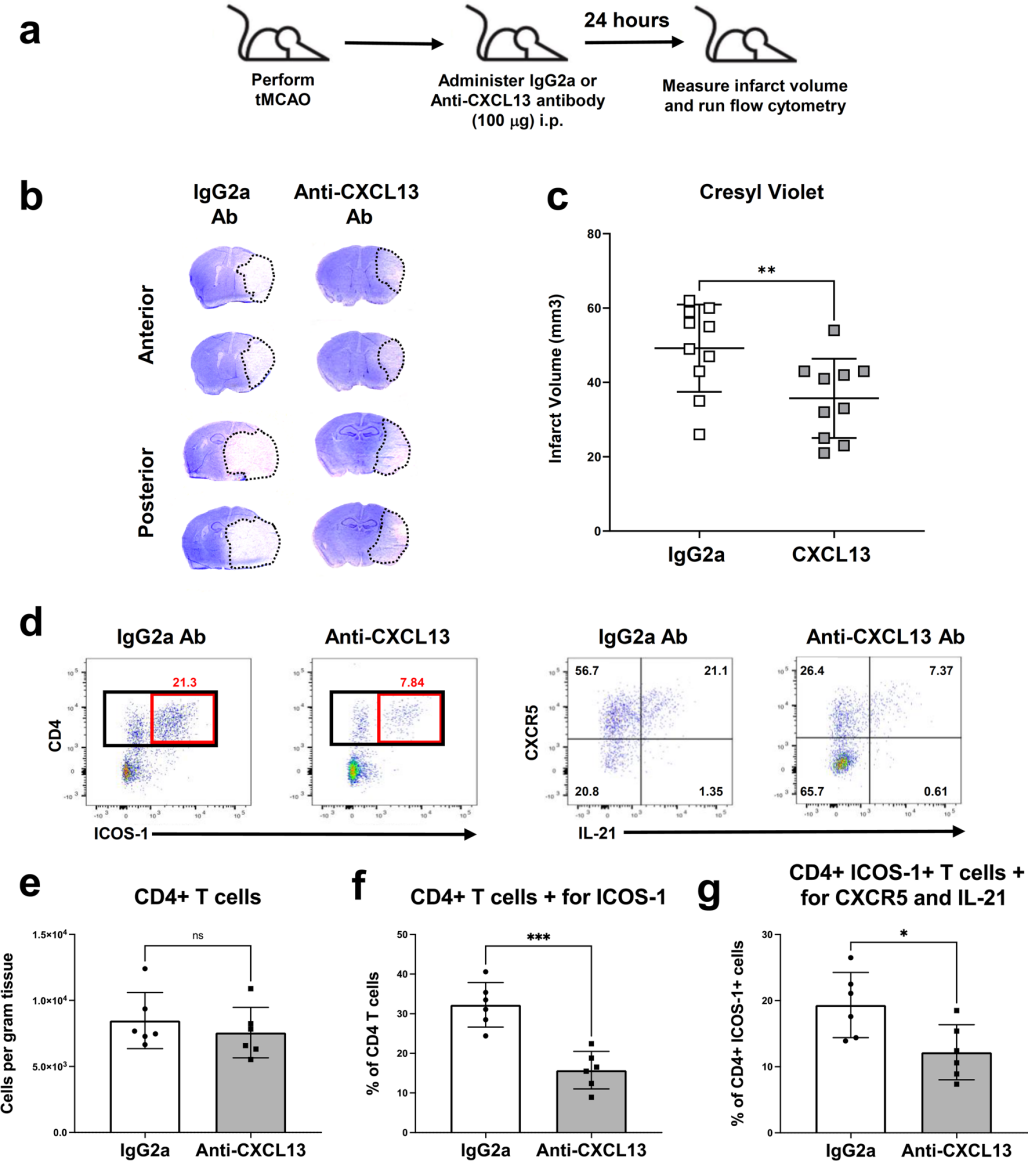
Administration of anti-CXCL13 antibody reduces infarct volume and inhibits the infiltration of T_FH_ cells into the brain following tMCAO. **a** Experimental design for administration of IgG2a and anti-CXCL13 antibody (100 μg) i.p. **b** Representative cresyl violet images to assess ischemic brain damage at 24 h tMCAO in IgG2a-treated and anti-CXCL13 antibody (100 μg)-treated mice. **c** Quantification of infarct volumes (mm^3^) at 24 h tMCAO in IgG2a-treated and CXCL13 antibody-treated mice (*n* = 10). **d** Representative flow cytometry gating of CD4+ ICOS-1+ and subsequent CXCR5+ IL-21+ cells from the ipsilateral brain of IgG2a isotype control-treated or anti-CXCL13 antibody-treated mice at 24 h following tMCAO. **e**–**g** Quantification of CD4+ T cells (**e**), % of CD4 T cells+ for ICOS-1 (**f**), and % of CD4+ ICOS-1+ T cells+ for CXCR5+ and IL-21+ (**g**) from IgG2a isotype control-treated and anti-CXCL13 antibody-treated mice at 24 h tMCAO. (*n* = 6) Data are combined from three independent experiments. Data represent mean ± s.e.m. *P < 0.05, **P < 0.01, ***P < 0.001, ****P < 0.0001. Student’s t test with Mann–Whitney *U* test (**c, e–g**)

Given that CXCR5 expressing T_FH_ cells migrate to the ischemic brain following tMCAO, we tested whether inhibiting CXCL13 could reduce T_FH_ cell recruitment and protect the brain after injury. To that end, we performed tMCAO on WT mice, administered anti-CXCL13 antibody (100 μg) i.p. or IgG2a isotype control within 5 min of reperfusion and evaluated the infarct volume at 24 h after tMCAO (Fig. 3a). Representative cresyl violet images and associated quantifications to depict the infarcted brain area revealed that anti-CXCL13 antibody treatment reduced the amount of ischemic brain damage at 24 h post-tMCAO (Fig. 3b, c).

We next performed flow cytometry on ipsilateral brains harvested from anti-CXCL13-treated and control-treated 24-h tMCAO mice to evaluate whether CXCL13 inhibition resulted in a decrease of IL-21 producing T_FH_ cell recruitment to brain. Representative gating and quantifications revealed that the anti-CXCL13 antibody treatment did not reduce total CD4+ T cell recruitment to the ischemic brain (Fig. 3e), but did reduce the amount of T_FH_ cells that infiltrated the ischemic brain at 24 h post-tMCAO compared to the IgG2a isotype control treatment (Fig. 3f, g). These data support that anti-CXCL13 can preferentially inhibit T_FH_ recruitment to the ischemic brain and reduce their impact on acute ischemic brain injury.

### CXCR5+ CD4+ T cells and CXCL13+ vessels localize to human acute stroke infarcts

**Fig. 4:**
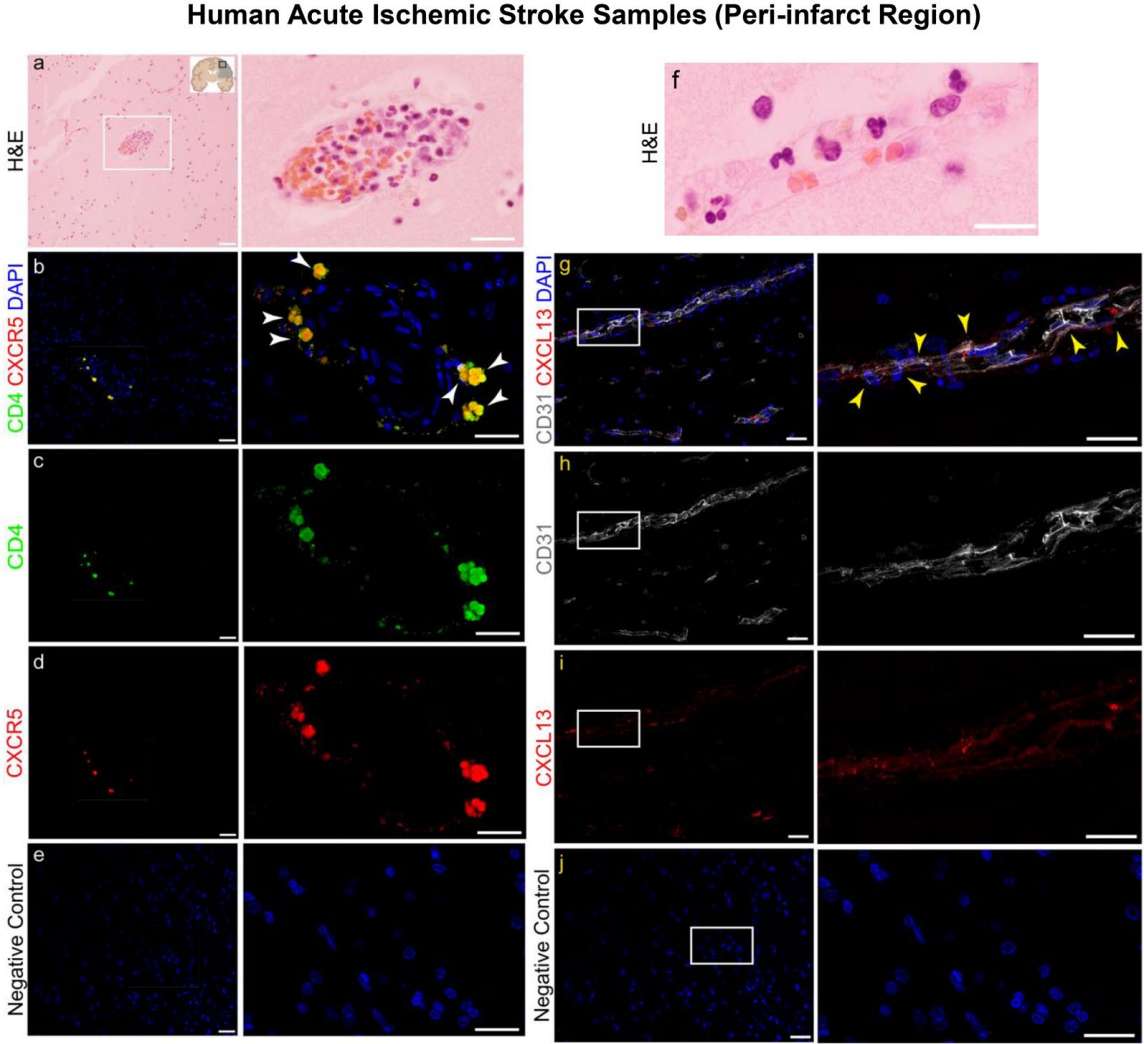
CXCR5+ CD4+ T cells and CXCL13+ vessels localize to human acute stroke infarcts. **a** Human brain section stained with hematoxylin and eosin (H&E) depicting immune cell cluster within the peri-infarct region of acute ischemic stroke patient brain. **b**–**e** Representative IHC staining of human acute stroke brain section with CD4 (green), CXCR5 (red), and DAPI (blue) (**b**), CD4 only (**c**), CXCR5 only (**d**), and negative control staining with secondary only and DAPI (**e**). **f** Human acute stroke tissue stained with H&E depicting an immune cell containing vessel within the brain. **g**–**j** Representative IHC staining of human acute stroke brain section with CD31 (white), CXCL13 (red), and DAPI (blue) (**g**), CD31 only (**h**), CXCL13 only (**i**), and negative control staining with secondary only and DAPI (**j**). Scale bar = 50 μm

Given our data obtained in mice, we explored whether we could observe signatures of T_FH_ cell recruitment to the acute human ischemic brain tissue. We first performed hematoxylin and eosin (H&E) staining, which revealed the presence of immune cell clusters in the peri-infarct region of these samples **(**Fig. 4a). Further staining identified CXCR5+ CD4+ T cells within those immune cell clusters, which are signature markers of T_FH_ cells (Fig. 4b–e). Moreover, we also identified CXCL13+ CD31+ vessels in the human ischemic brain tissue (Fig. 4f–j). These data are congruent with our immunostaining in mice and suggest that mechanisms related to the contribution of T_FH_ cells to acute stroke pathology in mice may apply to humans as well.

### IL-21R is upregulated in the ipsilateral brain hemisphere of mice 24 h after tMCAO in vivo and in human stroke brains

**Fig. 5:**
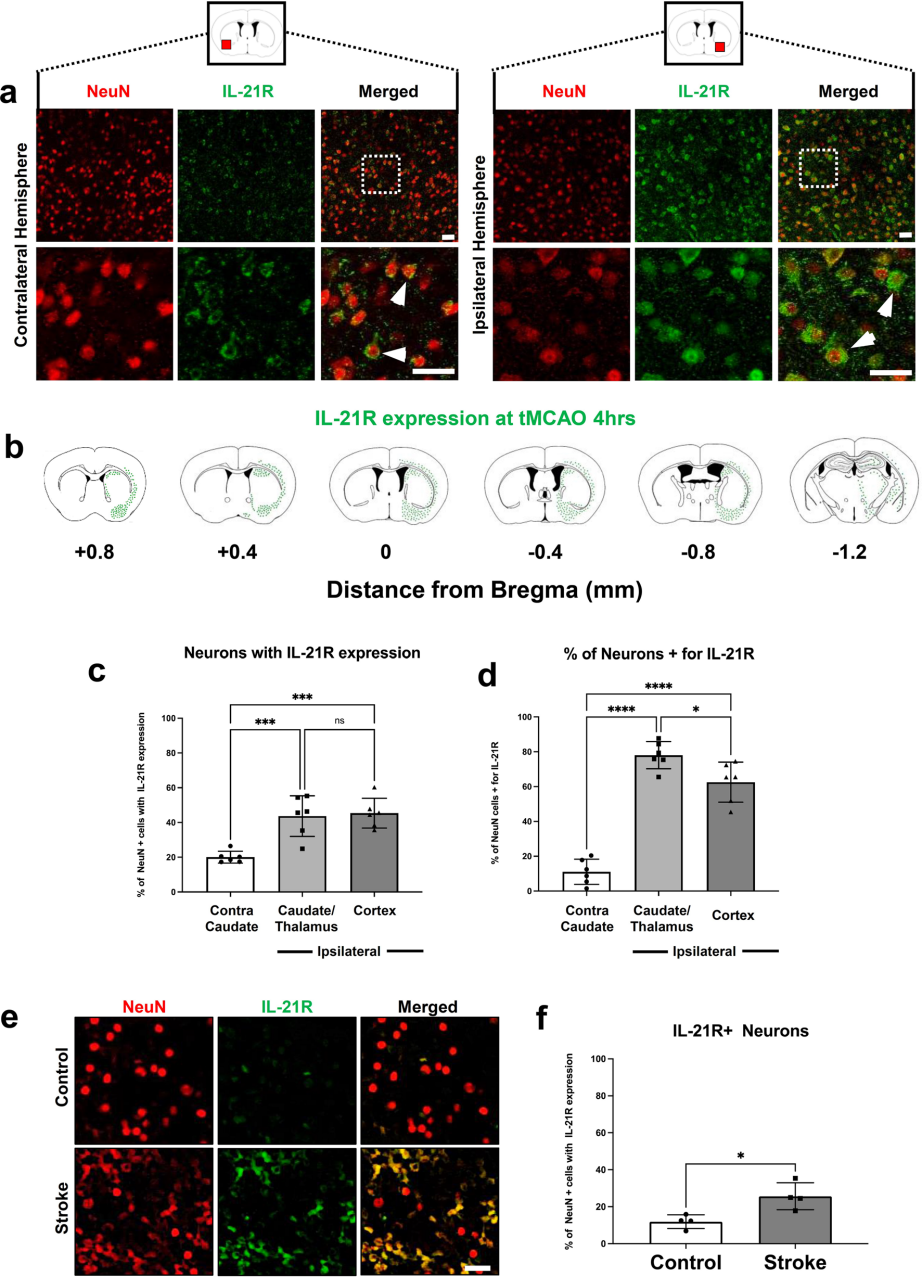
IL-21R is upregulated in the ipsilateral brain hemisphere of mice 24 h after tMCAO in vivo and in human stroke brains. **a** Low (top panels) and high (bottom panels) magnification representative IHC images stained with NeuN (red), IL-21R (green), and merged of contralateral brain hemisphere (left) and ipsilateral brain hemisphere (right) from mice 4 h after tMCAO. **b** Map of IL-21R/NeuN double-positive cells across coronal planes of mouse brains at 4 h tMCAO. Higher density of green dots represents a higher number of double-positive cells (*n* = 6). **c** Quantification of the percentage of NeuN-positive cells that are positive for IL-21R in the contralateral caudate, ipsilateral caudate/thalamus and ipsilateral cortex (*n* = 6). **d** Quantification of the proportion of neurons expressing IL-21R (relative co-localization per cell) in the contralateral caudate, ipsilateral caudate/thalamus and ipsilateral cortex (*n* = 6). **e** High magnification representative IHC images stained with NeuN (red), IL-21R (green), and merged in human acute stroke brains (top) and control brains (bottom). **f** Quantification of the percentage of NeuN-positive cells that are positive for IL-21R in human stroke and control stroke brain. (*n* = 4) Scale bar = 15 μm. White arrows indicate NeuN+ IL-21R+ cells. Murine data are combined from four independent experiments. Data represent mean ± s.e.m. *P < 0.05, **P < 0.01, ***P < 0.001, ****P < 0.0001. n.s. = not significant. One-way ANOVA followed by Dunn’s post hoc test (**c, d**) and Student’s *t* test with Mann–Whitney *U* test (**f**)

Given that we were able to clarify the mechanism for how IL-21 producing T_FH_ cells infiltrate the ischemic brain, our next question was to identify the cellular location of its cognate receptor, IL-21R, within the brain. IL-21R has been widely described to be present on immune cells within the periphery but limited studies have identified its presence on cells within the CNS [48, 49]. We stained brains harvested from mice 4 h post-tMCAO with NeuN and IL-21R in the ipsilateral brain hemisphere and contralateral hemisphere and observed that IL-21R is substantially more highly expressed in the ipsilateral hemisphere on neurons, while there is minimal expression in the contralateral hemisphere (Fig. 5a). These data reveal that IL-21R is upregulated in the infarcted brain hemisphere of mice in vivo. We then mapped out the co-localization of NeuN and IL-21R along coronal brain sections to get an understanding of the distribution of these double-positive cells (Fig. 5b). Quantification of the co-localization between NeuN and IL-21R along the coronal axis revealed that the ipsilateral peri-infarct areas had significantly more NeuN+ cells that were also positive for IL-21R (Fig. 5c). Moreover, we demonstrated that neurons in the ischemic hemisphere displayed greater overlap in co-localization with IL-21R compared to contralateral neurons implicating ipsilateral neurons express more IL-21R. This observation implicates that these neurons may display enhanced susceptibility to IL-21-mediated interactions (Fig. 5d). To test whether these NeuN+ IL-21R+ cells exist in ischemic brain tissue in our human samples, we stained human stroke tissue in the peri-infarct regions and in human control tissue. Representative images and quantifications reveal that these NeuN+ IL-21R+ cells not only exist, but also have a higher prevalence in human stroke tissue versus control tissue (Fig. 5e, f). Overall, these data demonstrate that IL-21R is upregulated after tMCAO on ischemic neurons.

### Administration of mIL-21 to primary neurons following OGD conditions induces Caspase 3/7-mediated apoptosis and activates JAK/STAT pathway

**Fig. 6:**
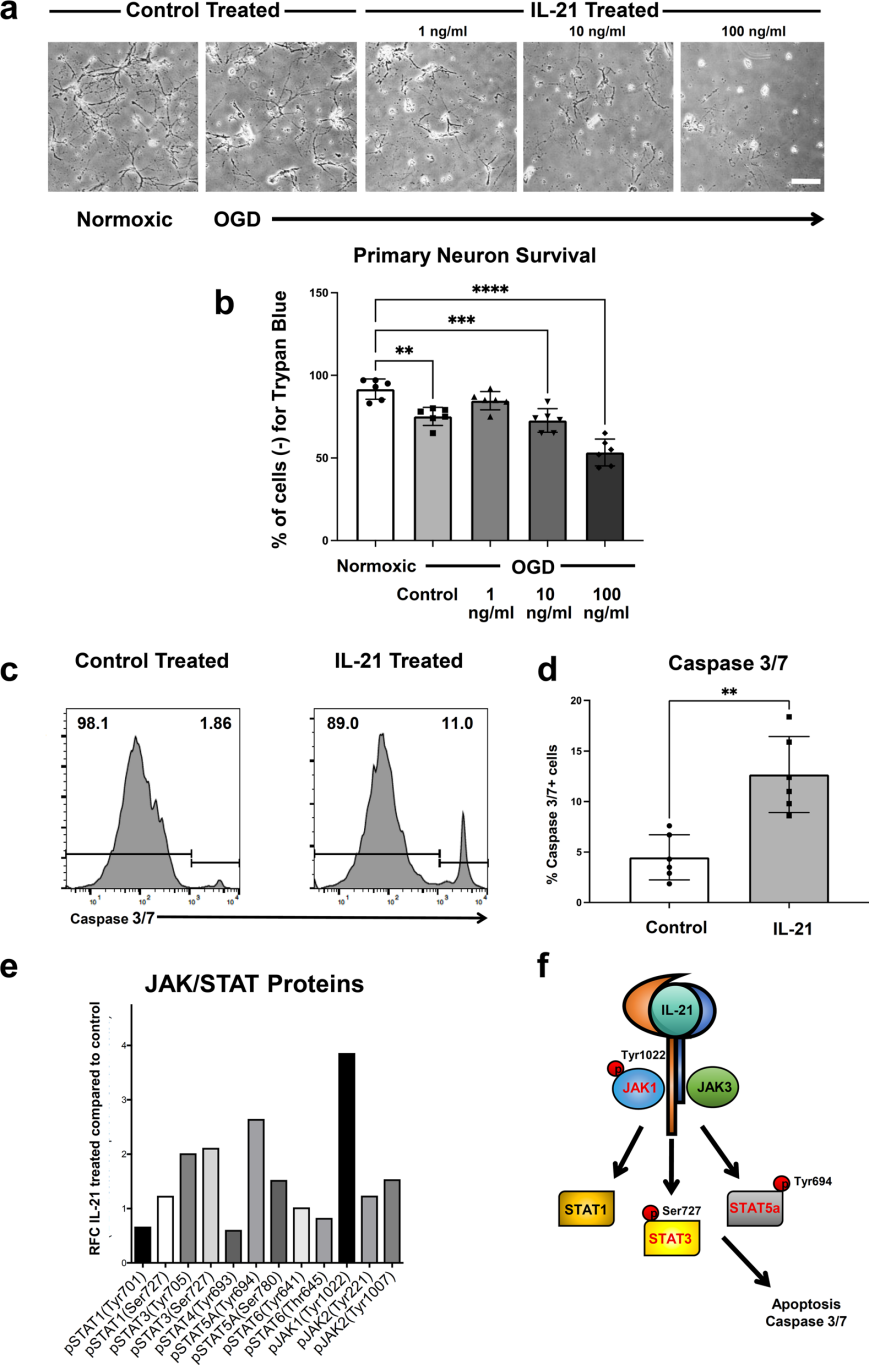
Administration of mIL-21 directly potentiates neuronal death following oxygen glucose deprivation conditions, induces Caspase 3/7-mediated apoptosis and activates JAK/STAT pathway in vitro. **a** Representative images of in vitro primary neurons under normoxic conditions (panel 1), oxygen glucose deprivation (OGD) conditions followed by exposure to complete media for 24 h (panel 2), OGD conditions followed by exposure to complete media with mIL-21 (1 ng/ml) for 24 h (panel 3), OGD conditions followed by exposure to complete media with mIL-21 (10 ng/ml) for 24 h (panel 4), and OGD conditions followed by exposure to complete media with mIL-21 (100 ng/ml) for 24 h (panel 5). Scale bar = 10 μm. **b** Quantification of primary neuron survival utilizing trypan blue cell exclusion assay following Normoxic conditions, OGD conditions followed by exposure to complete media for 24 h, as well as OGD conditions followed to exposure to complete media with mIL-21 (1 ng/ml, 10 ng/ml, and 100 ng/ml) for 24 h (*n* = 5). **c** Representative flow cytometry histograms of Caspase 3/7 activity on primary neurons treated with OGD conditions for 90 min followed by 24 h of normoxic conditions with control or mIL-21 (100 ng/ml) for 24 h. **d** Quantification of the percentage of neurons expressing Caspase 3/7 (*n* = 5). **e** Relative fold change of JAK/STAT pathway related phosphorylated proteins following IL-21 treatment compared to control-treated neurons in vitro. **f** Schematic of IL-21 activity on neurons. Data are combined from three independent experiments. Data represent mean ± s.e.m. Data represent mean ± s.e.m. *P < 0.05, **P < 0.01, ***P < 0.001, ****P < 0.0001. *n.s.* not significant. One-way ANOVA followed by Dunn’s post hoc test (**b**). Student’s t test followed by Mann–Whitney *U* test (**d**)

To test whether IL-21 directly affects neurons, we isolated primary neurons from murine cortices. We then exposed primary neurons to 90 min of oxygen glucose deprivation (OGD) or normoxic conditions with and without mIL-21 for 24 h in complete media to determine whether mIL-21 would affect neuronal morphology in hypoxic conditions. Representative images reveal that following OGD, exposure of neurons to complete media without mIL-21 had a baseline effect on the neuronal morphology but in a dose dependent manner, increasing concentrations of mIL-21 administration to OGD neurons correlated with decreased neuronal health (Fig. 6a). Quantification of the percentage of live neurons via trypan blue cell exclusion [50] confirmed that exposure of 90 min OGD neurons to 24 h of complete media with 10 ng/ml mIL-21 significantly reduced the percentage of live cells compared to 24 h of complete media alone. Additionally, increasing the exposure of 90 min OGD neurons to 24 h of complete media with 100 ng/ml mIL-21 significantly reduced the percentage of live cells even further (Fig. 6b). These data indicate that administration of mIL-21 dose-dependently and directly potentiates neuronal death following OGD in vitro.

Next, we performed a flow cytometry assay to evaluate the levels of caspase 3/7 activity following IL-21 treatment. Representative histograms and quantifications revealed that IL-21 treatment following OGD conditions leads to increased Caspase 3/7-mediated apoptosis in the neurons (Fig. 6c, d). IL-21 has been shown to activate the JAK/STAT pathway in peripheral immune cells in several disease contexts including rheumatoid arthritis [51] and lymphoma [52]. Yet to our knowledge, the role of IL-21 signaling on hypoxic neurons has not been evaluated. To evaluate the downstream signaling of IL-21R activation on neurons, we performed a JAK/STAT phosphorylation signaling array. This array revealed that several JAK/STAT related proteins get phosphorylated at specific sites in response to OGD and IL-21 treatment compared to OGD conditions only, particularly JAK1 at Tyrosine1022 (Tyr1022), STAT3 at Serine727 (Ser727), and STAT5A at Tyrosine694 (Tyr694), which could be the mechanism for IL-21-induced Caspase 3/7-mediated apoptosis in hypoxic neurons (Fig. 6e, f).

## Discussion

Here, we describe that following transient middle cerebral artery occlusion (tMCAO), CXCL13 is expressed by activated cerebral blood vessels, which contributes to the recruitment of CD4+ ICOS-1+ CXCR5+ IL-21 producing T follicular helper cells (T_FH_) into the brain during the reperfusion phase of the disease. Once these T_FH_ cells enter the brain, they can augment ischemic brain damage by producing IL-21 that directly targets IL-21R bearing neurons potentially through activation of the JAK/STAT pathway. Disruption of this pathway, by blocking chemotaxis of T_FH_ to the ischemic brain via antibody-mediated blocking of CXCL13 protects mice from tMCAO-mediated brain damage. Overall, these data identify a novel mechanism for how pro-inflammatory T_FH_ cells are recruited to the ischemic brain and elicit their damage during acute murine stroke and identify potential novel targets for stroke therapy.

The combination of CD4, ICOS-1, CXCR5, and IL-21 is a signature of T_FH_ cells, which have been identified to play an important role in helping B cells produce antibodies within B cell follicles of secondary lymph organs such as the lymph nodes, spleen and Peyer’s patches [39, 40]. Yet, while T_FH_ cells have been implicated to be involved in the pathology of CNS diseases [43, 53], to our knowledge, T_FH_ cells have never been identified in the ischemic brain. CXCL13 is considered to be a B lymphocyte-recruiting chemokine and is primarily expressed in lymphoid organs so why it gets upregulated in the ischemic brain is unknown. Given the literature implicating interactions with T_FH_ cells, CXCL13 and B cells, we measured B cells in the tMCAO brain early after tMCAO, but only identified a marginal subset of these cells 4 h and 24 h after injury compared to T cells (data not shown). Doyle et al. conducted a study to show that B cells contribute to delayed cognitive impairment following distal MCAO in mice [54] and our data demonstrate that T_FH_ cells are maintained in the ischemic brain days after tMCAO. Therefore, future studies aim to assess whether interaction between T_FH_ cells and B cells could mediate B cell-mediated neurotoxic responses at later time points after stroke.

CXCL13 has been observed to be expressed in the meninges of the CNS during autoimmune diseases and has emerged as a candidate as a therapeutic target for multiple sclerosis (MS) [27, 55–57] due to its role in myelinating glial cells [58]. During ischemic brain conditions however, CXCL13 seems to be mainly expressed on cerebral blood vessels [30]. Since glial cell death has been identified as a potential inducer of CNS autoimmunity [5, 59, 60] and blood–brain barrier breakdown is one of the primary events that occur following ischemic brain damage [61], CXCL13 may be a danger signal among several diseases that is upregulated to license peripheral immune cells for CNS entry through the damaged BBB. Evaluating how transgenic mice that have cell-specific deletion of CXCL13 respond to tMCAO and assessing whether T_FH_ cells infiltrate the CP or meninges could reveal additional mechanistic answers.

While we demonstrated ischemic brain protection in mice treated with anti-CXCL13 inhibition, it is noteworthy that the overall numbers of CD4 T cells in the damaged brain did not change relative to our control treatment. The presumably quelled phenotype of these potentially compensating CD4+ T cells in our treated group could be important for identifying their influence on stroke pathology. Moreover, while mice treated with the inhibitor were protected relative to control mice, treated mice still displayed considerable infarcts. Therefore, it will be interesting to test whether alternate inhibitors of post-stroke acute inflammation with distinct mechanisms utilized in conjunction with anti-CXCL13 treatment could compound its beneficial effects.

Since the signature cytokine of T_FH_ cells is IL-21, we searched for IL-21R expressing cells in the ischemic tissue and identified IL-21R expression on hypoxic neurons following tMCAO. At acute time points following ischemic conditions, our preliminary studies did not reveal any significant glial or endothelial cell expression of IL-21R in vivo or in vitro. While there is limited knowledge of IL-21R expression in the CNS, the expression of IL-21R has been widely reported on a myriad of cell types in the periphery and can play various roles that alter functional outcome depending on the target cell [48].

Recently, it was demonstrated that global deletion of IL-21R in mice is detrimental to stroke outcome in a different model (permanent MCAO) [62]. Given the double-edged sword nature of IL-21, it is possible that low levels of IL-21 could be protective on hypoxic neurons and high levels could be detrimental. To test this hypothesis, we dose-dependently administered mIL-21 to neurons and show that at 10 ng/ml, and 100 ng/ml, increasing concentrations of mIL-21 result in increasing amounts of neuronal death. Interestingly, at 1 ng/ml, we do see a protective effect on hypoxic neurons relative to untreated hypoxic neurons albeit it was not statistically significant**.** These studies highlight the importance of understanding the nuances of specific murine models of disease to properly evaluate how well therapies could translate to the clinic.

Elucidating how common gamma chain cytokine receptors like IL-21R initiate the activation of the JAK/STAT pathway can also be key in understanding how cytokines influence pathology. Often the same cytokine can elicit divergent responses depending on the timing and downstream effects on JAK/STAT related phosphorylation [63]. In our study, IL-21 had the largest effect on the phosphorylating at STAT3(Ser727), STAT5A(Tyr694), and JAK(Tyr1022) which led to caspase-mediated apoptosis in hypoxic neurons. Whether all or some of these phosphorylation events are required for IL-21-mediated apoptosis is unknown. Understanding the interplay between how different cytokines pleiotropically activate gamma chain cytokine receptors to influence stroke pathology through the JAK/STAT pathway could reveal more targeted therapeutic avenues for stroke.

## Conclusion

Our work is the first to identify a mechanism for how IL-21 producing T cells enter the ischemic brain and how these cells damage the inflamed tissue during the reperfusion phase of stroke. Future studies entail developing pharmacological tools that target neuronal IL-21R specifically, exploring the role of T_FH_ in the ischemic brain at later time points, and identifying the therapeutic window for CXCL13 inhibition. Moreover, revealing what regulates IL-21R expression on neurons following hypoxic conditions and understanding the role of the JAK/STAT pathway in stroke, can help elucidate why certain stroke patients may be susceptible to immune-mediated neuronal death. While we were able to demonstrate that some of features of our murine data are evident in human acute ischemic stroke tissue, we hope that findings from this work will lead to novel translational approaches for stroke patients.

## Data Availability

The data that support the findings of this study are available from the corresponding author upon request.

## Abbreviations

BBB: Blood–brain barrier
T_FH_: T follicular helper cells
JAK/STAT: Janus kinase/signal transducer and activator of transcription protein
MCAO: Middle cerebral artery occlusion
CNS: Central nervous system
CBF: Cerebral blood flood
PComA: Posterior communicating artery
ECA: External carotid artery
ICA: Internal carotid artery
tPA: Tissue plasminogen activator
ACK: Ammonium–chloride–potassium
RPM: Revolutions per minute
PBS: Phosphate-buffered saline
FACS: Flow cytometry staining buffer
CLN: Cervical lymph nodes
PFA: Paraformaldehyde
IL: Interleukin
CTL: Cytotoxic T lymphocytes
MMP: Matrix metallopeptidase
TNF: Tumor necrosis factor
WT: Wild type
